# Antioxidant Activity in Gilthead Seabream (*Sparus aurata* L.) Fed with Diet Supplemented with Moringa

**DOI:** 10.3390/antiox10091423

**Published:** 2021-09-07

**Authors:** Antonia M. Jiménez-Monreal, Francisco A. Guardiola, M. Ángeles Esteban, M. Antonia Murcia Tomás, Magdalena Martínez-Tomé

**Affiliations:** 1 Department of Food Science, Faculty of Veterinary, Regional Campus of International Excellence “Campus Mare Nostrum”, University of Murcia, 30100 Murcia, Spain; mamurcia@um.es (M.A.M.T.); mmtome@um.es (M.M.-T.); 2 CIBER: CB12/03/30038 Fisiopatología de la Obesidad y la Nutrición, CIBEROBN, Instituto de Salud Carlos III (ISCIII), 28029 Madrid, Spain; 3 Immunobiology for Aquaculture Group, Department of Cell Biology and Histology, Faculty of Biology, Regional Campus of International Excellence “Campus Mare Nostrum”, University of Murcia, 30100 Murcia, Spain; faguardiola@um.es (F.A.G.); aesteban@um.es (M.Á.E.)

**Keywords:** fish farm, fatty acid, Moringa polyphenol content, free radical scavenging

## Abstract

Gilthead seabream is bred mainly in fish farms in the Mediterranean Sea. One important factor responsible for the deterioration of fish quality is lipid oxidation. *Moringa oleifera* leaves have been described as having high antioxidant content. This work investigates the effect of dietary supplementation with Moringa leaves on the antioxidant activity of seabream. Gilthead seabream specimens were divided into four groups, the control group (fed a commercial diet) and three other groups fed diets enriched with Moringa (5%, 10% and 15%). The antioxidant capacity was measured by assays of free radical scavenging (OH·, H_2_O_2_, lipoperoxyl and ABTS), Rancimat test and linoleic acid system in muscle and skin of gilthead seabream, commercial diet, enriched diet and Moringa. Finally, the polyphenol content of Moringa and the fatty acid composition of seabream fed diets with and without Moringa were determined. Results showed an increase in antioxidant activity in gilthead seabream fed with diets enriched with a higher percentage of Moringa; therefore, Moringa could be considered a functional ingredient in diets for fish bred in fish farms and. The antioxidant potential of Moringa leaves could be mainly attributed to the presence of polyphenolic compounds.

## 1. Introduction

The close relationship between food and health has led to changes in consumer habits. There is a current trend of modification in feeding of animals to obtain healthy benefits for the consumer, as well as an increase in the nutritional quality of food, without altering its sensory characteristics. The feeding of animals with plants containing antioxidant compounds might serve to introduce the antioxidants into their body which, in turn will be consumed by the population [1,2].

There is a growing interest in reducing free radicals present in food that justifies the usefulness of natural antioxidants in order to improve quality, texture, colour, taste, nutritional value and increased shelf life of food. Natural antioxidants are mainly obtained from food such as fruits, vegetables, cereals, spices and traditional medicinal herbs and plants [3,4].

The Moringa (*Moringa oleífera* L.) is a tropical plant containing small or medium-sized perennial leaves. This plant is native to northern India and is a widely cultivated spice [5]. It is considered one of the world’s most useful trees as it has nutritional, medicinal and other beneficial properties [6,7].

Interest in the study of Moringa has been shown in various regions of the world where the population has nutritional problems. The consumption of the leaves, green pods and seeds of this plant has been promoted as a source of various nutrients [8]; leaf flour has actually been used as an alternative food source to combat malnutrition, particularly in children [9]. Moringa leaves also present a high content of antioxidants and antimicrobial activity due to the presence of ascorbic acid, flavonoids, phenolic compounds and carotenoids [10,11]. Recently, in vivo and in vitro experiments have demonstrated that moringa exhibits immunostimulatory [12], cytotoxic, bactericidal and antioxidant activities [13] in gilthead seabream (*Sparus aurata*, L), the species selected in this study. In addition, moringa has been reported to reduce stress biomarker levels in rabbits [14] and several fish species with particular interest in the aquaculture sector, such as Nile tilapia (*Oreochromis niloticus*) and common carp (*Cyprinus carpio*) [15,16].

Taking into account the recent increase in foods enriched with antioxidants and the growing interest in the scientific community of the benefits of the intake of foods with antioxidant properties, the aim of the proposed study is to assess the effect of diets enriched with Moringa, in the antioxidant activity of gilthead seabream, which was selected for being one of the most important aquaculture species in the Mediterranean Sea and, therefore, for human consumption.

## 2. Materials and Methods

### 2.1. Fish

Eighty (125.7 ± 9.5 g weight) specimens of the marine teleost gilthead seabream (*S. aurata*, L.) were obtained from a local farm (Murcia, Spain) and acclimatized for 2 weeks in recirculating seawater aquaria (250 L) at the Marine Fish Facility, University of Murcia. The water temperature was maintained at 20 ± 2 °C with a flow rate of 900 L/h and 28% salinity. The photoperiod was adjusted to 12 h light/12 h dark. Fish were fed with a commercial pellet diet (Skretting, Spain) at a rate of 2% body weight/day. Fish were allowed to acclimatise for 15 days before the start of the experimental trial. All experimental protocols were approved by the Ethical Committee of the University of Murcia and carried out in accordance with EU Directive 2010/63/EU [17] for animal experiments.

### 2.2. Experimental Design

Eighty fish were randomly distributed into four identical tanks (20 fish per group), where the following groups were established: (1) control, non-supplemented diet (0%); (2) 5%, diet supplemented with 5 g/100 g of Moringa leaves; (3) 10%, diet supplemented with 10 g/100 g of Moringa leaves; (4) 15%, diet supplemented with 15 g/100 g of Moringa leaves. The fish were fed twice daily for a four-week period at a rate of 2% body weight per day. Twenty specimens from each group were sampled at the end of the trial. All specimens were sacrificed with an overdose of MS-222 (Sandoz, Holzkirchen, Germany, 1000 mg L^−1^ water).

### 2.3. Diet

The commercial diet (control diet, Skretting, Burgos, Spain) was mixed with a suitable amount of Moringa and compacted into granules, thus obtaining diets supplemented with 5%, 10% and 15% Moringa. The prepared diet was subjected to a drying process and stored at 4 °C. The composition of commercial diet (D-2 Optibream AE 1P) was crude protein (48%), fat (18%), ash (6.3%) and cellulose (3.6%).

### 2.4. Sample Preparation

For sample preparation, seabream muscle was extracted and homogenized with a Moulinex Turbo blender (Model Q48) and stored until analysis at −80 °C in a Thermo Electron Corporation freezer (Model ULT790-5-V34). Using the same process, we proceeded with the skin of the gilthead seabream.

In addition, diets supplemented with Moringa, commercial diet, Moringa leaves and an additive propyl gallate (PG), commonly used in the food industry as an antioxidant, were analysed.

### 2.5. Assays of Free Radical Scavenging and Antioxidant Activity

#### 2.5.1. Hydroxyl Radical Scavenging

The deoxyribose assay is used to detect possible scavengers of hydroxyl radicals, formed by a mixture of ascorbate and FeCl3-EDTA. The products resulting from the hydroxyl radical (OH·) attack on deoxyribose were measured with thiobarbituric acid [18].

#### 2.5.2. Measurement of Total Antioxidant Activity by the TEAC Assay

This method is based on inhibition by antioxidants of the 2,2-azino-bis-(3-ethylbenzothiazoline-6-sulfonic) (ABTS) radical cation. The Trolox Equivalent Antioxidant Capacity (TEAC) assay measures the ability of antioxidants to quench the ABTS cation in both lipophilic and hydrophilic environments by comparing their scavenging capacity to that of Trolox [19].

#### 2.5.3. Peroxidation of Phospholipid Liposomes

The ability of samples to inhibit lipid peroxidation was tested by using ox brain phospholipid liposomes. The extent of peroxidation was measured in accordance with Jiménez et al. [20].

#### 2.5.4. Rancimat Test for Oxidative Stability

Sample preparation in the Rancimat test consisted of macerating the different samples (seabream muscle, seabream skin, diets supplemented with Moringa, commercial diet, Moringa leaves) at 10% (*w*/*w*) concentration, while the propyl gallate was used at the permitted commercial concentration of 100 µg/g with sunflower oil for 3 h at room temperature before analysis. After 3 h of maceration, the Rancimat apparatus began to measure conductivity. Oxidative stability, evaluated by the Rancimat method at 110 °C (Metrohm model 743, Herisan, Switzerland), reflects resistance to development of rancidity in oils/fats. Results were expressed by the protection factor (PF), calculated from the induction period (IP, in hours), the time until a critical point of oxidation is reached [20].

#### 2.5.5. Determination of Antioxidant Activity in a Linoleic Acid System

This method, which is used to determine the antioxidant activity of samples during storage at unfavourable temperatures (40 °C), measures the inhibition of linoleic acid autoxidation [21].

### 2.6. Determination of Fatty Acid Composition

The fatty acid composition was determined by gas chromatography according to the official method AOAC [22]. Methylation of fatty acids was carried out according to AOAC [23]. Fatty acid composition of the muscle of gilthead sea bream fed with and without diet supplemented with 10% *Moringa oleifera* for two and four weeks was analysed. The FAMEs were separated by GC (Varian 3900 GC, Varian, Inc., Walnut Creek, CA, USA) equipped with a fused silica capillary column (HP-88, 100 m 9 0.25 mm 9 0.20 lm film), split injector and flame ionization detector fitted with a Galaxie Chromatography Data System (Version 1.9.3.2 software). The result of each fatty acid was expressed as %. The total omega-3 fatty acid (mg/100 g) was determined by CG and total omega-6 fatty acids (mg/100 g) were determined by adding all omega-6 fatty acids.

### 2.7. Determination of Total Polyphenol Contents

The total polyphenol content of food was determined using the Folin–Ciocalteau method [24]. In summary, 0.2 mL of an extracted sample was mixed with 1 mL of Folin–Ciocalteau reagent previously diluted in distilled water (1:10) and 0.8 mL of 7.5% (*w*/*v*) sodium carbonate. Absorbance was measured after 30 min at 765 nm on a Spekol 10 spectrophotometer, Carl Zeiss Jena, Germany. Results are expressed as grams (g) of gallic acid equivalents (GAE) per 100 g of dried Moringa (g GAE/100 g DM).

### 2.8. Statistical Analysis

All measurements were performed on three replicates. Results are expressed as mean ± standard deviation, sd. Data were statistically analysed by one-way analysis of variance (ANOVA) to determine differences between groups. Statistical analyses were conducted using SPSS 27.0 and differences were considered statistically significant when *p* ≤ 0.05.

## 3. Results

### 3.1. Antioxidant Activity

Antioxidant activity was analysed in the muscle and skin samples of seabream fed for four weeks with diets supplemented with Moringa (5%, 10% and 15%) and a commercial diet. The results of the antioxidant activity evaluated by lipid peroxidation, hydroxyl radical scavenging and TEAC are shown in Table 1. In the lipoperoxyl radical inhibition, a significant increase (*p* < 0.05) in activity of muscle was observed (43% inhibition) in sea bream fed with a diet supplemented with 15% Moringa. Results showed a very high hydroxyl radical scavenging capacity in all samples of muscle, values around 80%, but no significant differences were observed as regards seabream fed with a commercial diet; similar behaviour was observed in the TEAC assay. As for antioxidant activity evaluated in the skin, samples were good lipoperoxyl radical scavengers and did not show significant differences (*p* < 0.05). Lower values of hydroxyl radical scavenging were observed in skin samples with respect to muscle, values around 65%, without significant differences observed. All samples also exhibited antioxidant activity when ascorbate was omitted from the reaction mixture (primary antioxidant) as they were able to directly scavenge the hydroxyl radical generated.

**Figure 1:**
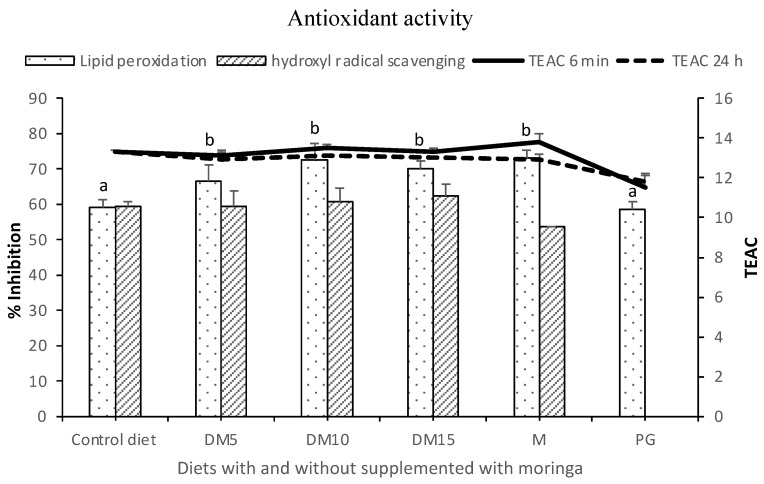
Antioxidant activity of different diets (diet base: non-supplemented diet; 5%, diet supplemented with 5 g/100 g of *Moringa oleifera* (MO); 10%, diet supplemented with 10 g/100 g of MO); and 15%, diet supplemented with 15 g/100 g of MO) and MO leaves compared with common food additive antioxidant, propyl gallate (PG) evaluated by different assays—hydroxyl radical scavenging, lipid peroxidation and TEAC. All determinations were performed in triplicate and the columns represent the mean ± standard deviation. Values are expressed as percentage of scavenging of peroxidation lipid and of the hydroxyl radicals and TEAC value. Control diet, commercial diet; DM5, diet with 5% MO; DM10, diet with 10% MO; DM15, diet with 15% MO; M, MO leaves; PG, propyl gallate. TEAC is the micromolar concentration of a Trolox solution showing the antioxidant capacity equivalent to the dilution of the substance under investigation at 6 min and 24 h. Different superscript letters indicate significant differences (ANOVA, *p* < 0.05).

Figure 1 shows the antioxidant activity of diets used in feeding of seabream, evaluated by lipid peroxidation, deoxyribose and TEAC assays. Moringa leaves and the antioxidant additive commonly used in the food industry, PG, were also evaluated. Results show a high antioxidant activity of diets supplemented with Moringa (66.5%, 72.6% and 70.2% percentages of scavenging in diets supplemented with 5%, 10% and 15%, respectively), with significant differences as regards the commercial diet, and even show values higher than those obtained with PG (*p* < 0.05) in the lipid peroxidation assay.

The Rancimat assay is used to determine whether the antioxidant activity of samples resists heating at high temperatures [25].

**Figure 2:**
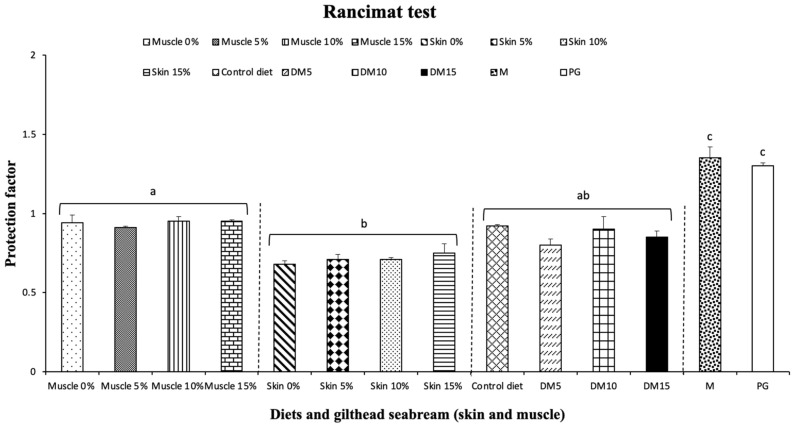
Antioxidant activity of diets with and without supplemented *Moringa oleifera* (MO) (5, 10 and 15%), MO leaves and samples of gilthead seabream (muscle and skin) fed with and without supplemented diet with MO for 4 weeks evaluated by Rancimat test, compared with activity of common food antioxidant additive, PG. All determinations were performed in triplicate and the columns represent the mean of PF ± standard deviation. Muscle 0%, muscle of Gilthead seabream fed control diet without MO; Muscle 5%, muscle of Gilthead seabream fed diet with 5% MO; Muscle 10%, muscle of Gilthead seabream fed diet with 10% MO; Muscle 15%, muscle of Gilthead seabream fed diet with 15% MO; Skin 0%, skin of Gilthead seabream fed control diet without MO; Skin 5%, skin of Gilthead seabream fed diet with 5% MO; Skin 10%, skin of Gilthead seabream fed diet with 10% MO; Skin 15%, skin of Gilthead seabream fed diet with 15% MO; PG, propyl gallate; Control diet, commercial diet; DM5, diet with 5% MO; DM10, diet with 10% MO; DM15, diet with 15% MO; M, MO leaves. Different superscript letters indicate significant differences (ANOVA, *p* < 0.05).

Figure 2 shows the antioxidant activity of diets with and without supplemented Moringa (5, 10 and 15%), Moringa leaves and samples of gilthead seabream (muscle and skin) fed with and without supplemented diet with Moringa for 4 weeks, evaluated by Rancimat test, compared to the activity of a common food antioxidant additive. The protection factor expresses the oxidative stability of sunflower oil with analysed samples. The Moringa leaves show the highest value (1.35) (*p* < 0.05), which protects slightly by increasing induction time even higher than the antioxidant additive (PG) (1.30). Results of the seabream skin samples were lower (*p* < 0.05) than those obtained for seabream muscle (*p* < 0.05). However, all samples presented values lower than 1, with samples fed with diets supplemented with Moringa showing a low oxidative protection.

**Figure 3:**
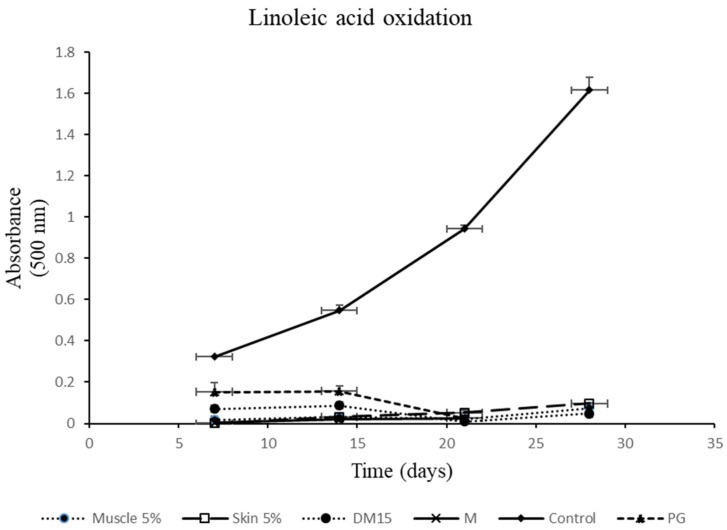
Evolution of the absorbance at 500 nm for the oxidation of linoleic acid, for 28 days of storage at 40 °C, in the presence of diet supplemented with 15 g/100 g of MO (15%), of samples of gilthead seabream (muscle and skin) fed with diet supplemented with 5 g/100 g of MO (5%) and of MO leaves, for four weeks, compared with a common food additive antioxidant, PG. All determinations were performed in triplicate and values shown are mean ± standard deviation. MO, *Moringa oleifera*; Level 1, M (MO leaves); Level 2, Muscle 0%, Muscle 5%, Muscle 10%, Muscle 15% (muscle of Gilthead seabream fed control diet without MO and diet supplemented with 5%, 10% and 15%); Skin 0%, Skin 5%, Skin 10%, Skin 15% (skin of Gilthead seabream fed control diet without MO and diet supplemented with 5%, 10%, 15%); Control diet (commercial diet); DM5, DM10, DM15 (diet supplemented with 5%, 10%, 15% MO) and PG (propyl gallate); Level 3, control (without samples).

The total antioxidant activity was determined by linoleic acid system. This method is used to determine antioxidant activity during storage at unfavourable temperatures (40 °C) measuring the inhibition of linoleic acid autoxidation during storage, which was measured at 500 nm. Figure 3 shows the evolution of absorbance at 500 nm for the oxidation of linoleic acid, during 28 days of storage, in the presence of diet supplemented with 15 g/100 g of Moringa (15%), of samples of gilthead seabream (muscle and skin) fed with diet supplemented with 5 g/100 g of Moringa (5%) and of Moringa leaves, for four weeks, compared with a common food additive antioxidant, PG. Results are classified into three levels. In the first level, the Moringa leaves to which very high antioxidant activity is attributed are presented; in the second, all diets and sea bream samples (muscle and skin) fed with a diet supplemented with Moringa and the antioxidant additive PG were included. Finally, the control sample is included in the third level, with significant differences, compared to the rest of samples (*p* < 0.05).

### 3.2. Fatty Acid Composition of Gilthead Seabream

Moringa has a high content of unsaturated fatty acids, oleic acid above all (70%) and linoleic acid [7,26,27]. Therefore, we were able to determine the composition of fatty acids in seabream fed with a diet supplemented with Moringa. Table 2 shows the fatty acid composition of gilthead seabream fed with and without supplemented diet with Moringa 10% for 2 and 4 weeks. Results showed a significant increase (*p* < 0.05) in values of oleic acid (1.20% to 1.75%) and linoleic acid (0.51% to 0.64%), such as total omega 6 fatty acids (0.53% to 0.66%), in the seabream samples fed for two weeks with diets enriched with Moringa at 10%.

### 3.3. Total Phytochemical Contents of Leaves and Seed from Moringa

Additionally, the content of phenolic compounds in Moringa was analysed, which accounts for a large part of its antioxidant activity. Table 3 shows the polyphenol content of different leaves and seeds extracts from Moringa. It was observed that, in the leaf, the content of total polyphenols was much higher than that found in the seed, with a mean value of 4.3 g GAE/100 g DM (*p* < 0.05). In all samples (leaf and seed), the highest content of polyphenols found was carried out by extraction with methanol, the lowest content being in water extracts (*p* < 0.05). Finally, the extracts with methanol/water (80/20) showed similar polyphenol contents compared to water extracts, without significant differences.

## 4. Discussion

The diet of gilthead seabream is based on molluscs, crustaceans and small fish; therefore, fishmeal-based feed is administered in fish farms. Some studies have shown that ingredients based on vegetable crops are the most promising alternative feed ingredients, particularly if produced by terrestrial agriculture in the local region and could contribute directly to the sustainability and cost-effectiveness of fish farming. Vegetable protein meals with high protein content are those most widely tested as substitutes for fish meal [28].

Moringa, according to Gopalakrishnanb et al. [29], is grown for its nutritious pods, edible leaves and flowers and can be utilized as food, medicine, cosmetic oil, or forage for livestock. Moringa seed extract also has the ability to eliminate heavy metals from water and wastewater, as a low cost biosorbent [30]. Moringa has been studied for its health properties, antimicrobial and antioxidant properties, attributed to the numerous bioactive components, including vitamins, phenolic acids (chlorogenic acid, cafeic acid, etc.), flavonoids (kaempferol, etc.), isothiocyanates, tannins and saponins, present in significant amounts in various components of the plant [31]. In our study, we have observed a high content of polyphenols, especially in Moringa leaf extracts; thus, we have used the leaves to formulate diets enriched with Moringa to feed seabream. Other authors [32] also determined the content of polyphenols in Moringa leaves, obtaining values between 3.6 and 4.7 g GAE/100 g DM. Furthermore, Rocchetti et al. [33] obtained three different Moringa leaf extracts by using methanol, methanol: water 50:50 *v*/*v* and ethyl acetate, revealing a large abundance of flavonoids and phenolic acids. Each extraction solvent was found to promote different extraction efficiency, the highest total phenolic content being recorded in methanol 100% leaf extracts (31.84 mg phenolic equivalents/g DM). Similar results were observed by Nobossé et al. [34], where the polyphenol content in Moringa leaves was higher in methanol extract (3.91 g GAE/100 g DM), followed by ethanol extract (3.32 g GAE/100 g DM) and, finally, water extract (3.20 g GAE/100 g DM). Our results are in agreement with these studies.

We observed different behaviour when analysing antioxidant activity evaluated in the gilthead seabream samples fed a diet supplemented with Moringa. In the case of lipid peroxidation inhibition, gilthead seabream muscle samples show a significant increase in activity when supplemented with 10 and 15% Moringa. This behaviour is also observed when analysing diets, since antioxidant activity is high in those supplemented with 5%, 10% and 15% Moringa, even higher than the activity of PG; therefore, it could be used for the prevention of lipid peroxidation in diets.

Sharmin et al. [35] found similar results in terms of oxidative stability when analysing the effect of diets enriched with Moringa to produce value-added broiler meat. Salem et al. [36] also observed reduced MDA levels in rabbits which were given diets enriched with Moringa, compared to those fed without Moringa; Ahmed et al. in Nile Tilapia [37] observed a significant reduction in MDA levels and a large increase of enzymatic activities of SOD, CAT, GSH, GPx when fed with dietary Moringa supplementation.

In addition, oxidative stability in oils using substances such as antioxidants is important, since it allows delaying degradation time of fats and oils and extends their useful life. In this regard, results obtained in the Rancimat or oxidative stability test, where a longer period of induction of oxidation was observed in the Moringa leaves analysed, suggest they have a high protection factor against oxidation. Nascimento et al. [38], also studied the oxidative stability of the different parts of Moringa (leaves, flowers and seeds), obtaining the greatest stability for leaves due to their higher content of phenolic compounds, justifying their greater antioxidant capacity. However, supplemented diets were not as protective as Moringa, possibly due to the doses used. Guardiola et al. [39], revealed that the dietary supplement of fenugreek seeds in gilthead seabream could enhance antioxidant behaviour in a dose-dependent way. Other studies have reported on fish fed with herbal dietary supplements and the variations observed are in accordance with the nature of the substances, doses and duration [40,41].

Siddhuraju and Becker [42] examined water, aqueous methanol and aqueous ethanol extracts of freeze-dried leaves of Moringa for radical scavenging capacities and antioxidant activities. They observed that the extracts of Moringa leaves inhibited 89.7–92.0% peroxidation of linoleic acid after incubation for 14 days and showed radical scavenging activity in a manner dependent on the dose in the β-carotene–linoleic acid system. These data are consistent with the high antioxidant activity obtained against the autoxidation of linoleic acid in studied samples. Flavonoids are among the most potent plant antioxidants, as they possess an o-diphenol group in ring B, a 2–3 double bond conjugated with the 4-oxo function and hydroxyl groups in positions 3 and 5. Flavonoids are very effective scavengers of peroxyl radicals and are also chelators of metals and inhibit the Fenton and Haber–Weiss reactions, which are important sources of active oxygen radicals. Flavonoids also retain their free radical scavenging capacity after forming complexes with metal ions. In this context, based on the chemical reactions discussed above, the presence of flavonoids, such as quercetin, kaempferol and other phenolic compounds, in various Moringa leaf extracts could be involved in the inhibition of peroxidation [42,43]. For this reason, the antioxidant effect of Moringa added to the diet is more pronounced in a lipid medium, especially peroxyl radicals.

It must be taken into account that the lipid profile of some fish species is characterized by the presence of long-chain polyunsaturated fatty acids, such as eicosapentaenoic acid (EPA) and docosahexaenoic acid (DHA), susceptible to oxidative deterioration [44]. Various studies have shown that seabream muscle tissue is especially sensitive to changes in its fatty acid composition through diet [45]. Based on the foregoing, the muscle of seabream fed with diets enriched with Moringa shows a significant increase in levels of both linoleic and oleic acid; therefore, it is important to consider the antioxidant effect of Moringa as a protective factor against oxidative damage

## 5. Conclusions

Supplementation in the diet of seabream with Moringa supposes an increase in the antioxidant activity of the muscle; therefore, it could be considered a functional ingredient in fish bred in fish farms, thus providing food with improvement in oxidative stability. The antioxidant potential of *Moringa oleifera* leaves could be mainly attributed to the presence of polyphenolic compounds. These results are encouraging enough to continue with subsequent studies on the use of Moringa as an antioxidant, although it would be necessary to optimize the appropriate concentration in animal supplementation in order for the antioxidant activity to be more effective.

## Figures and Tables

**Table 1:** Antioxidant activity of samples of gilthead seabream (muscle and skin) fed with and without supplemented diet with *Moringa oleifera* (MO) (5, 10 and 15%) for 4 weeks evaluated by different assays (lipid peroxidation, hydroxyl radical scavenging and TEAC). All determinations were performed in triplicate and values shown are mean ± standard deviation. Different superscript letters indicate significant differences (*p* < 0.05).

	Lipid Peroxidation	Hydroxyl Radical Scavenging		TEAC ^1^	
Samples of Gilthead Seabream	% Scavenging	% Scavenging	Absorbance (A_532 nm_) Omit ASC ^2^	6 min	24 h
None (control)			0.133 ± 0.009 ^b^		
Muscle					
0%	29.2 ± 1.6 ^a^	78.85 ± 1.3 ^a^	0.045 ± 0.004 ^a^	12.1 ± 0.5 ^b^	12.5 ± 0.4 ^a^
5%	33.1 ± 2.3 ^ab^	83.9 ± 3.9 ^a^	0.093 ± 0.077 ^ab^	10.2 ± 0.8 ^a^	12.0 ± 0.6 ^a^
10%	34.9 ± 1.3 ^b^	81.6 ± 4.6 ^a^	0.088 ± 0.008 ^ab^	11.2 ± 0.9 ^ab^	12.0 ± 0.6 ^a^
15%	43.0 ± 0.9 ^c^	80.7 ± 0.2 ^a^	0.069 ± 0.026 ^ab^	12.1 ± 0.4 ^b^	12.5 ± 0.7 ^a^
Skin					
0%	70.9 ± 3.8 ^d^	58.5 ± 7.2 ^b^	0.105 ± 0.004 ^ab^	15.5 ± 0.0 ^c^	14.4 ± 0.0 ^b^
5%	65.7 ± 2.5 ^d^	67.2 ± 7.2 ^b^	0.093 ± 0.038 ^ab^	15.0 ± 0.5 ^c^	14.8 ± 0.4 ^b^
10%	64.0 ± 8.1 ^de^	61.5 ± 5.2 ^b^	0.107 ± 0.058 ^ab^	15.5 ± 0.3 ^c^	15.1 ± 0.8 ^b^
15%	68.6 ± 2.1 ^d^	63.9 ± 3.3 ^b^	0.126 ± 0.031 ^b^	15.6 ± 0.1 ^c^	14.4 ± 0.1 ^b^

^1^ TEAC is the micromolar concentration of a Trolox solution showing the antioxidant capacity equivalent to the dilution of the substance under investigation at 6 min and 24 h. ^2^ When ascorbic acid was omitted, absorbance values were lower than control. 0%, gilthead seabream (muscle and skin) fed control diet; 5%, gilthead seabream (muscle and skin) fed diet with 5% MO; 10%, gilthead seabream (muscle and skin) fed diet with 10% MO; 15%, gilthead seabream (muscle and skin) fed diet with 15% MO.

**Table 2:** Fatty acid composition of gilthead seabream fed with and without supplemented diet with *Moringa oleifera* (MO) 10% for 2 and 4 weeks. All determinations were performed in triplicate and values shown are mean ± standard deviation.

		2 Weeks			4 Weeks		
	Fatty Acids	Without Moringa	10% Moringa	*p*	Without Moringa	10% Moringa	*p*
SFA							
	Myristic (14:0)	0.11 ± 0.03	0.12 ± 0.03		0.24 ± 0.09	0.25 ± 0.10	
	Pentadecenoic (15:0)	0.10 ± 0.04	0.01 ± 0.01		-	0.02 ± 0.01	
	Palmitic (16:0)	0.53 ± 0.20	0.61 ± 0.12		1.14 ± 0.41	1.23 ± 0.60	
	Heptadecenoic (17:0)	0.01 ± 0.01	0.02 ± 0.02		0.01 ± 0.01	0.01 ± 0.01	
	Stearic (18:0)	0.10 ± 0.04	0.12 ± 0.03		0.21 ± 0.09	0.23 ± 0.10	
	Arachidic (20:0)	0.02 ± 0.02	0.03 ± 0.01		0.01 ± 0.01	0.01 ± 0.01	
	Behenic (22:0)	0.01 ± 0.01	0.01 ± 0.01		0.02 ± 0.01	0.02 ± 0.01	
	Tricosanoic (23:0)	0.02 ± 0.01	0.03 ± 0.02		0.05 ± 0.02	0.05 ± 0.01	
	Lignoceric (24:0)	0.08 ± 0.03	0.10 ± 0.04		0.19 ± 0.07	0.20 ± 0.10	
MUFA							
	Myristoleic (14:1n-5)	-	-		0.01 ± 0.01	0.01 ± 0.01	
	Pentadecenoic(15:1n-5)	-	-		0.02 ± 0.01	-	
	Palmitoleic (16:1n-7)	0.16 ± 0.08	0.18 ± 0.08		0.35 ± 0.06	0.38 ± 0.05	
	Heptadecenoic (17:1n-7)	0.01 ± 0.01	0.01 ± 0.01		0.02 ± 0.01	0.02 ± 0.01	
	Oleic (18:1n-9)	1.20 ± 0.30	1.75 ± 0.21	*	2.21 ± 0.85	2.31 ± 0.96	
	Eicosenoic (20:1n-9)	0.11 ± 0.05	0.14 ± 0.05		0.22 ± 0.11	0.22 ± 0.11	
	Erucic (22:1n-9)	0.05 ± 0.02	0.07 ± 0.03		0.12 ± 0.03	0.12 ± 0.03	
	Nervonic (24:1n-9)	0.02 ± 0.03	0.02 ± 0.01		0.04 ± 0.03	0.03 ± 0.03	
PUFA							
Omega-6							
	Linoleic (18:2n-6) cis	0.51 ± 0.10	0.64 ± 0.10	*	1.03 ± 0.45	1.01 ± 0.27	
	g-linolenic (18:3n-6)	-	-		0.02 ± 0.01	0.02 ± 0.01	
	Eicosadienoic (20:2n-6)	0.01 ± 0.01	0.02 ± 0.02		0.04 ± 0.03	0.04 ± 0.03	
	Arachidonic (20:4n-6)	-	-		0.01 ± 0.01	0.01 ± 0.01	
	Docosadienoic (22:2n-6)	-	-		0.01 ± 0.01	0.01 ± 0.01	
	Total omega-6	0.53 ± 0.01	0.66 ± 0.02	*	1.10 ± 0.02	1.08 ± 0.01	
Omega-3							
	Linolenic (18:3n-3)	0.09 ± 0.02	0.11 ± 0.05		0.21 ± 0.10	0.21 ± 0.10	
	Eicosatrienoic (20:3n-3)	0.04 ± 0.03	0.04 ± 0.03		0.09 ± 0.02	0.08 ± 0.05	
	Docosahexaenoic (22:6n-3)	0.16 ± 0.05	0.20 ± 0.06		0.36 ± 0.03	0.36 ± 0.03	
	Total omega-3	0.30 ± 0.02	0.36 ± 0.01		0.66 ± 0.02	0.66 ± 0.01	

* Significant differences (*p* < 0.05).

**Table 3:** Polyphenol content of different leaves and seed extracts from *Moringa oleifera*. All determinations were performed in triplicate and values shown are mean ± standard deviation. Different superscript letters indicate significant differences (*p* < 0.05).

Samples Moringa	Total Polyphenols (g GAE ^1^/100 g DM)
Leave	
Water extract	4.03 ± 0.13 ^b^
Methanol extract	4.56 ± 0.15 ^a^
Methanol/water extract (80/20)	4.20 ± 0.21 ^ab^
Seed	
Water extract	0.51 ± 0.17 ^d^
Methanol extract	1.18 ± 0.20 ^c^
Methanol/water (80/20)	0.76 ± 0.19 ^d^

^1^ GAE, gallic acid equivalent.

## Data Availability

All data are contained in this article.
